# *Cratoxylum formosum* (Jack) Dyer ssp. *pruniflorum* (Kurz) Gogel. (Hóng yá mù) extract induces apoptosis in human hepatocellular carcinoma HepG2 cells through caspase-dependent pathways

**DOI:** 10.1186/1749-8546-9-12

**Published:** 2014-04-07

**Authors:** Apiyada Nonpunya, Natthida Weerapreeyakul, Sahapat Barusrux

**Affiliations:** 1 Graduate school, Faculty of Pharmaceutical Sciences, Khon Kaen University, Khon Kaen 40002, Thailand; 2 Center for Research and Development of Herbal Health Products (CRD-HHP), Khon Kaen University, Khon Kaen 40002, Thailand; 3 Faculty of Pharmaceutical Sciences, Khon Kaen University, Khon Kaen 40002, Thailand; 4 Centre for Research and Development of Medical Diagnostic Laboratories (CMDL), Khon Kaen University, Khon Kaen 40002, Thailand; 5 Faculty of Associated Medical Sciences, Khon Kaen University, Khon Kaen 40002, Thailand

## Abstract

**Background:**

*Cratoxylum formosum* (Jack) Dyer ssp. *pruniflorum* (Kurz) Gogel. (Hóng yá mù) (CF) has been used for treatment of fever, cough, and peptic ulcer. Previously, a 50% ethanol-water extract from twigs of CF was shown highly selective in cytotoxicity against cancer cells. This study aims to investigate the molecular mechanisms underlying the apoptosis-inducing effect of CF.

**Methods:**

The cytotoxicity of CF was evaluated in the human hepatocellular carcinoma (HCC) HepG2 cell line in comparison with a non-cancerous African green monkey kidney epithelial cell line (Vero) by a neutral red assay. The apoptosis induction mechanisms were investigated through nuclear morphological changes, DNA fragmentation, mitochondrial membrane potential alterations, and caspase enzyme activities.

**Results:**

CF selectively induced HepG2 cell death compared with non-cancerous Vero cells. A 1.5-fold higher apoptotic effect compared with melphalan was induced by 120 μg/mL of the 50% ethanol-water extract of CF. The apoptotic cell death in HepG2 cells occurred *via* extrinsic and intrinsic caspase-dependent pathways in dose- and time-dependent manners by significantly increasing the activities of caspase 3/7, 8, and 9, decreasing the mitochondrial membrane potential, and causing apoptotic body formation and DNA fragmentation.

**Conclusions:**

CF extract induced a caspase-dependent apoptosis in HepG2 cells.

## Background

Hepatocellular carcinoma (HCC) is the fifth leading cancer worldwide and the third cause of cancer death [1] with a poor prognosis owing to the ineffectiveness of therapy [2]. The treatment options for HCC patients, such as surgical resection and liver transplantation [2], are only appropriate for the early stages of tumor development. A second primary site can be caused by a malignant clone even if the first cancer site has been diagnosed and resected [1]. Chemoprevention is considered to be another important strategy for both primary and secondary HCC prevention of HCC by phytochemicals or non-nutritive plant compounds. The development and research of new anticancer agents has decreased HCC-associated mortality [2].

Some anti-cancer agents induced apoptosis of cancer cells [3]. Apoptotic cancer cells are characterized by morphological changes, including chromatin condensation, membrane blebbing, and apoptotic body formation, which are related to DNA fragmentation observed as a ladder on gel electrophoresis [2]. Apoptosis comprises two major pathways, the intrinsic and extrinsic pathways [4]. The caspase enzymes are proteases playing an important role in the classification of apoptosis. Several studies have screened potential compounds that can trigger apoptosis *via* measurement of caspase activity [5-7]. Currently, anticancer drugs such as cisplatin, paclitaxel, adriamycin, and melphalan have been reported to eradicate cancer *via* the induction of apoptosis [5,7-9].

*Cratoxylum formosum* (Jack) Dyer ssp. *pruniflorum* (Kurz) Gogel. (CF) (Hóng yá mù or commonly known as “Kuding Tea” in Southwest mainland China [10] and “Tiew kon” in Thailand [11]) is widely found in Southeast Asia [12] and belongs to the family of Guttiferae. Five other *Cratoxylum* species were found in Thailand and Southwest China, namely *Cratoxylum arborescens*, *Cratoxylum cochinchinense*, *Cratoxylum maingayi*, *Cratoxylum sumatranum* ssp. *neriifolium*, and *Cratoxylum formosum* (Jack) Dyer [13].

CF has been used in Chinese medicine for treatment of fever, cough, stomach ache, flatulence, diarrhea, food poisoning, internal bleeding, and peptic ulcer [10,13]. It has also been used as a diuretic and for its tonic effects [10]. CF produces various secondary metabolites including xanthones, anthraquinones [10,14], triterpenoids, flavonoids [13-16], and phenolic compounds [6]. These compounds are widely found in plants and some of them have been shown to exert cytotoxic effects toward some cancer cell lines and other biological activities such as antibacterial effects and antimicrobial effects [10].

Fractions obtained from dichloromethane crude extract of CF roots was reported to exhibit anticancer activity against MCF-7 human breast cancer cells, human cervical cancer HeLa cells, colon cancer HT-29 cells, and human oral cancer KB cells [13]. The cytotoxic effect of the CF extract was selectively high against human leukemia cancer cells (U937) compared with normal cells (Vero). Our group previously reported the apoptotic effect of CF on U937 cells, based on observations of DNA laddering and nuclear morphological changes [8]. This study aims to investigate the molecular mechanisms underlying the apoptosis-inducing effect.

## Materials and methods

### Reagents and chemicals

The organic solvents used for extraction were of analytical grade and obtained from Fisher Scientific (Loughborough, UK) and Labscan (Bangkok, Thailand). The reagents used in the cell assays were of molecular biological grade. Dulbecco’s modified Eagle’s medium (DMEM), fetal bovine serum (FBS), penicillin, and streptomycin were purchased from GIBCO® (Invitrogen Corporation, Carlsbad, CA, USA). Standard reagents, melphalan, and 4′,6-diamidino-2-phenylindole (DAPI) were obtained from Sigma-Aldrich Inc. (St. Louis, MO, USA). Dimethylsulfoxide (DMSO) was purchased from United States Biological (Salem, MA, USA). The reagents for cytotoxicity testing included neutral red (NR) and sodium bicarbonate (NaHCO_3_). We used a FlexiGene DNA Kit (Qiagen GmbH, Germany), isopropyl alcohol biotechnology grade (Bio Basic Inc., USA), and methanol analytical grade (BDH, England). Agarose molecular grade was purchased from Bio-Rad (Hercules, CA, USA). We purchased a 100 bp + 1.5 kb DNA ladder stained with loading dye from SibEnzyme Ltd. (Russia). Caspase-Glo® 3/7, 8, and 9 Assay Kits were purchased from Promega (Madison, WI, USA). We obtained 3,3′-dihexyloxacarbocyanine iodide (DiOC6) from Sigma-Aldrich Inc. Pure concentrated hydrogen peroxide (35%, v/v) was purchased from Qrec Co. Ltd. (New Zealand).

### Cell culture

An African green monkey kidney cell line (Vero) at passage 26 and a human HCC cell line (HepG2) at passage 40 were maintained in DMEM supplemented with 10% FBS and 1% (w/v) penicillin/streptomycin. All cells were cultured at 37°C in a humidified atmosphere containing 5% CO_2_.

### Plant material and extraction

Twigs of CF were collected in 2008 from the northeastern region of Thailand. The plants were visually authenticated according to taxonomy [17] and voucher specimens (TT-OC-SK-862) were deposited at the Medicinal Herbarium of the Faculty of Pharmaceutical Sciences, Khon Kaen University, Thailand. Briefly, 1 kg of dried plants was cut and macerated with 6 L of 50% ethanol and water for 7 days. The solvent was filtered, distilled *in vacuo* by a rotary evaporator below 40°C, and freeze-dried, yielding a 3.78% (w/w) crude extract. The crude extract was stored at 4°C in an airtight container. The stock solution of CF extract was freshly prepared and dissolved in 100% DMSO, and the concentration of DMSO in all experiments was restricted to ≤ 1% to maintain the cytotoxicity at < 10%.

### Cytotoxicity assay

Cell viability was measured by an NR colorimetric assay. Human HepG2 cells and normal African green monkey kidney Vero cells were used as cell models to determine the percent cytotoxicity. Briefly, cells (4 × 10^6^ cells/mL) were treated with CF extract at various concentrations (10, 25, 50, 100, 250, and 500 μg/mL) and the standard anticancer drug melphalan as a positive control, while the untreated groups were exposed to fresh medium at equal volumes to those used for the treated groups. After 24 h of exposure, the cells were incubated for 2 h with NR dye (50 μg/mL) dissolved in DMEM, and then washed with phosphate-buffered saline (PBS; pH 7.4). Finally, the cells were lysed with 0.33% HCl/isopropanol. A microplate reader (Tecan, France) was used to measure the absorbances of NR dye in the viable cells at 520 and 650 (reference) nm. The percentage of cell cytotoxicity of the CF extract was calculated as described by Machana *et al.*[18] and compared with that of melphalan. The selectivity index (SI) was calculated to determine the selective cancer cell line cytotoxicity [9]. The SI of the test compound was compared with the IC_50_ for the Vero cells divided by the IC_50_ for the HepG2 cells.

### Apoptosis induction assay

#### *DAPI staining assay*

Cells (1 × 10^6^ cells/well) were treated with CF extract and the melphalan for 24 h at 2 × IC_50_, and the morphological changes of the induced apoptotic cells were determined by DAPI staining. The concentrations of CF extract and melphalan used in the following apoptosis experiments were 120 and 80 μg/mL, respectively. The cells were fixed in pure methanol at 4°C for 10 min, and then stained with DAPI (1 μg/mL) at room temperature in the dark. The excess dye was removed and glycerin was added to the cells with PBS at a 1:1 ratio and mixed well. Next, the cells were fixed on a slide and the apoptotic bodies observed at 40× objective magnification under an inverted fluorescence microscope (Eclipse TS100; Nikon, UK). The percentage of apoptotic cells was calculated as described by Machana *et al.*[18]. The average% apoptotic cells were calculated from three independent wells with 10 eye views in each well.

#### *DNA fragmentation assay*

HepG2 cells (1 × 10^6^ cells/well) were treated with 120 μg/mL of CF extract or 80 μg/mL of melphalan for 24 h, harvested, and washed three times with sterile PBS. Each cell suspension was transferred to a microcentrifuge tube (1.5 mL) and centrifuged (DAIHAN scientific, Korea) at 300 × *g* for 5 min, and the cell pellet was collected. A FlexiGene DNA kit was used to extract the DNA in accordance with the manufacturer’s instructions. Subsequently, 2 μg of DNA was analyzed by electrophoresis (Gibthai, Taiwan) in a 1.8% agarose gel containing 0.1% (v/v) ethidium bromide. The DNA fragments were analyzed by an InGenius L Gel Documentation system (Syngene, MD, USA) and an image of the electrophoresis gel was taken.

#### *Caspase activity assay*

HepG2 cells (4 × 10^6^ cells/mL) were seeded in a 96-well plate, incubated overnight, and then treated with CF extract at 0, 30, 60, 120, and 240 μg/mL for 24 h (caspase 3/7 activity test) or 12 h (caspase 8, 9 activity test). In addition, HepG2 cells were treated with CF extract (120 μg/mL) at 0, 3, 6, 12, and 24 h for caspase 3/7 activity and 0, 3, 6, 9, and 12 h for caspase 8 and 9 activity). The enzyme activity of caspase 3/7, 8, and 9 was determined by Caspase-Glo® 3/7, 8, and 9 Assay Kits in accordance with the manufacturer’s instructions. The caspase activities were expressed as relative luminescence units (RLU) in direct proportion to the luminescent light and were detected by a microplate luminometer (SpectraMax Gemini X, Sandy, UT, USA). Blank determinations were performed on a test plate containing only sample and medium. All of the experiments were performed in triplicate.

#### *Detection of mitochondrial membrane potential (MMP)*

Cells (1 × 10^6^ cells/mL) were seeded into a 24-well plate, incubated at 37°C for 24 h, and then treated with 0, 30, 60, or 120 μg/mL of CF extract for 12 or 24 h. Concentrated hydrogen peroxide (35%, v/v) was used as a positive control. Next, the cells were washed, harvested by trypsinization, transferred to a microcentrifuge tube, and stained with a lipophilic fluorogenic dye, DiOC6, at 40 nM for 30 min in the dark at 37°C. After the incubation, the cells were immediately analyzed by flow cytometry (BD FACSCanto II, San Jose, CA, USA) for incorporation of the fluorochrome.

### Determination of phenolics in the CF extract

The total phenolic content was determined by the Folin-Ciocalteu reagent as described by Kaisoon *et al.*[19]. Briefly, 300 μL of CF extract was mixed with 2.25 mL of Folin-Ciocalteu reagent (10-fold dilution with distilled water). After standing at room temperature for 5 min, 2.25 mL of saturated NaHCO_3_ (60 g/L) solution was added to the mixture. After 90 min, the absorbance of the reaction mixture was measured at 725 nm by a UV-spectrophotometer (Shimadzu, Japan). The results were expressed as milligrams of gallic acid equivalents per 1 g of dried sample (mg GAE/g dry weight).

### Statistical analysis

The data were represented by the mean ± standard deviation (SD) (n = 3). The statistical significance of differences for multiple-group comparisons was evaluated by one-way ANOVA followed by a Tukey honestly significant difference using SPSS Windows version 11.5 (SPSS, USA). Evaluation with a two-tailed Student’s *t*-test was also performed for two-group comparisons. Values of *P* < 0.05 were considered statistically significant.

## Results

### CF extract induced human HCC cell death

**Figure 1:**
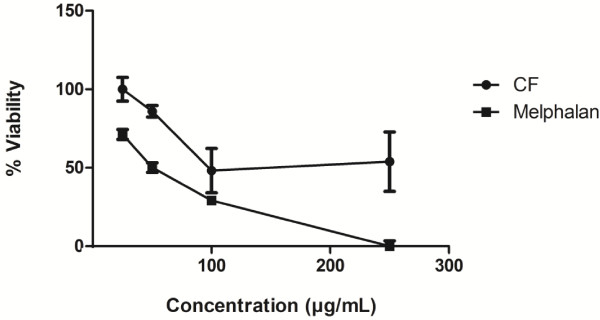
The HepG2 viability profile after treated with melphalan or CF for 24 h (n = 3).

The cytotoxicity (Table 1) and growth inhibition (Figure 1) results of the crude CF extract compared to melphalan showed cytotoxicity with a 50% inhibitory concentration (IC_50_ ± SD) of 55.9 ± 10.6 μg/mL for HepG2 cells while no effect on normal Vero cells at concentrations up to 500 μg/mL. Melphalan exhibited an IC_50_ of 37.7 ± 9.8 μg/mL for HepG2 cells, but showed low selectivity (SI = 1.6). The CF extract showed high selectivity (SI = 8.9) against HepG2 cells with a minimum effect on normal Vero cells. The CF extract possessed potential cytotoxic activity toward the HCC cell line *in vitro*.

**Table 1:** Cytotoxicity and selectivity index of 50% ethanol-water extract of CF on HepG2 cells compared with Vero cells

**Sample**	**Part used**	**IC**_**50**_ **± SD (μg/mL)**		**Selectivity index (SI)**^**c**^
		**HepG2**	**Vero**	
*Cratoxylum formosum* (Jack) Dyer ssp. *pruniflorum* (Kurz) Gogel.	Twig	55.9 ± 10.6	Inactive^b^	8.9
Melphalan^a^		37.7 ± 9.8	59.9 ± 3.2	1.6

Data expressed as the mean ± SD of three determinations.

^a^Standard anticancer drug.

^b^IC_50_ > 500 μg/mL is considered inactive.

^c^SI referred to Selectivity Index, when SI value > 3 indicates high selectivity.

### CF extract induced apoptosis

#### *CF caused nuclear morphological changes in HepG2 cells*

**Figure 2:**
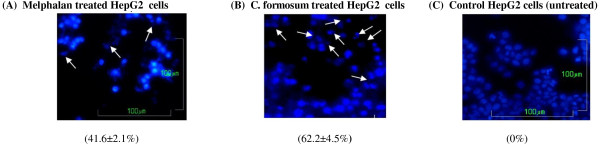
**Nuclei morphological changes and percentage of apoptotic cells in HepG2 cells (A) after treatment with 80 μg/mL of melphalan, (B) 120 μg/mL of CF, and (C) in the control or untreated HepG2 cells.** Apoptotic bodies are indicated by white arrows. Pictures captured using an inverted fluorescent microscope at 40 × objective magnification.

Images of nuclear DNA staining by DAPI in untreated (control) and treated HepG2 cells were shown in Figure 2. Nuclei with condensed chromatin and apoptotic bodies, characteristic of late-stage apoptosis [20], were observed in HepG2 cells incubated with the CF extract and the melphalan. The results revealed that the CF extract caused a higher percentage of apoptosis (62.2 ± 4.5%) as compared to melphalan alone (41.6 ± 2.1%). Apoptotic bodies were not observed in the untreated HepG2 cells.

#### *CF induced apoptotic DNA fragmentation in HepG2 cells*

**Figure 3:**
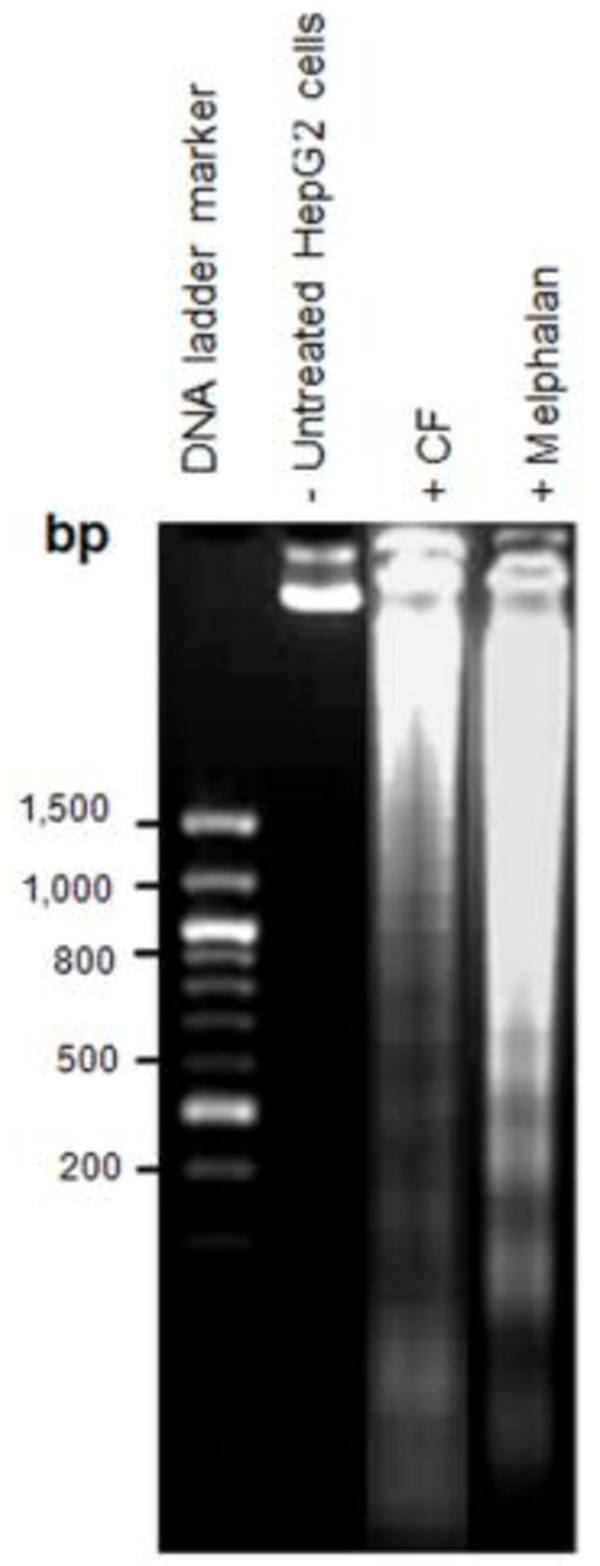
DNA fragmentation in HepG2 cells treated with 80 μg/mL of melphalan or 120 μg/mL of CF extract for 24 h.

Apoptotic cell death leads to regulated nuclear fragmentation by endonuclease enzymes in a controlled manner that involves DNA fragmentation [20]. During the apoptotic process, the release of endonuclease enzymes associated with DNA fragmentation in apoptotic cells results in DNA laddering, which can be observed by agarose gel electrophoresis [21]. This phenomenon specifically indicates that treated cancer cells have undergone late-stage apoptosis [22]. Figure 3 shows that treatment of HepG2 cells with 120 μg/mL of CF extract led to DNA laddering after 24 h of exposure, and were similar to those for cells treated with melphalan alone.

### Apoptosis induction mechanisms of the CF extract

#### *CF induces apoptosis in HepG2 cells via activation of caspase activity*

**Figure 4:**
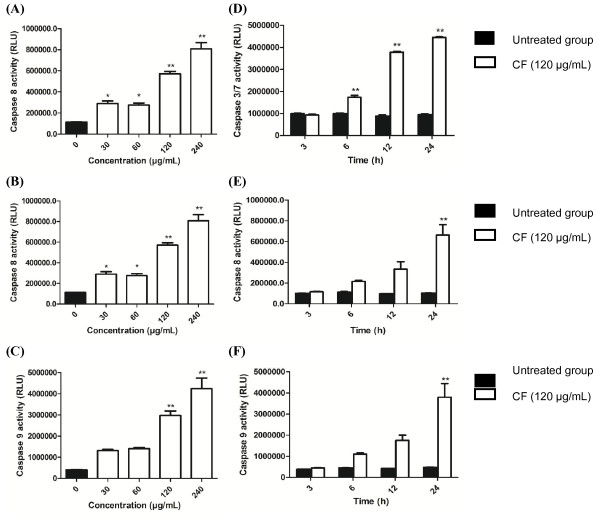
**Activation of caspase-3/7, -8 and -9 in the HepG2 treated with the CF extract.** Relative luminescent units (RLU) of **(A)** caspase 3/7, **(B)** 8 and **(C)** 9, respectively, after HepG2 cells exposed to CF extract at various concentrations for 12 (for caspase 8, 9) and 24 (for caspase 3) h. **(D)**, **(E)**, and **(F)** represent the caspase 3/7, 8, 9 activity after being treated with CF extract (120 μg/mL) at various points. Values represent a mean ± SD, based on triplicates. *P < 0.05, **P < 0.001 significantly different from untreated cells.

Apoptotic cell death occurs *via* major extrinsic and intrinsic pathways. Caspases 3/7, 8, and 9 are the major apoptotic caspases. Activation of caspase 8 indicates the extrinsic pathway, while activation of caspase 9 indicates the intrinsic pathway. When cells undergo apoptosis through either the extrinsic or intrinsic pathway, caspase 3 activity is activated at the final step prior to cell death [5-7]. The apoptosis induced in HepG2 cells by the CF extract through caspase activity was initially observed at 30 μg/mL after 6 h of incubation (Figure 4). The CF extract increased caspases activity with dose and time dependent manner. At 24 h the CF extract at all concentration tested significantly increased caspase 3/7 from the untreated cells (*P* < 0.001, one-way ANOVA). The cells treated with 30, 60, 120, and 240 μg/mL significantly increased caspase 8 activity (*P* = 0.018, *P* = 0.029, *P* < 0.001, *P* < 0.001, respectively, one-way ANOVA). However, no significant difference of caspase 9 activity was observed (one-way ANOVA) between the group of cells treated with 30 and 60 μg/mL and the untreated cells (*P* = 0.150 and *P* = 0.100). Whereas the group of cells treated with 120, and 240 μg/mL significantly increased caspase 9 activity compared to the untreated cells (*P* < 0.001, one-way ANOVA).

At 3 h the CF extract (120 μg/mL) did not significantly increase caspase 3/7 (*P* = 0.885), caspase 8 (*P* = 1.000), and caspase 9 activity (*P* = 1.000) when compared to the control (one-way ANOVA). The CF extract (120 μg/mL) significantly increased caspase 3/7 at 6, 12, and 24 h (*P* < 0.001, one-way ANOVA). The CF extract at the same concentration did not significantly increased caspase 8 at 6 and 9 h (*P* = 0.597, and *P* = 0.07) but significantly increase caspase 8 activity at 12 h (*P* < 0.001, one-way ANOVA). Moreover, the CF extract at the same concentration did not significantly increased caspase 9 at 6 and 9 h (*P* = 0.568, and *P* = 0.079) but significantly increase caspase 9 activity at 12 h (*P* < 0.001, one-way ANOVA).

#### *CF induced apoptosis in HepG2 cells through the mitochondrial pathway*

**Figure 5:**
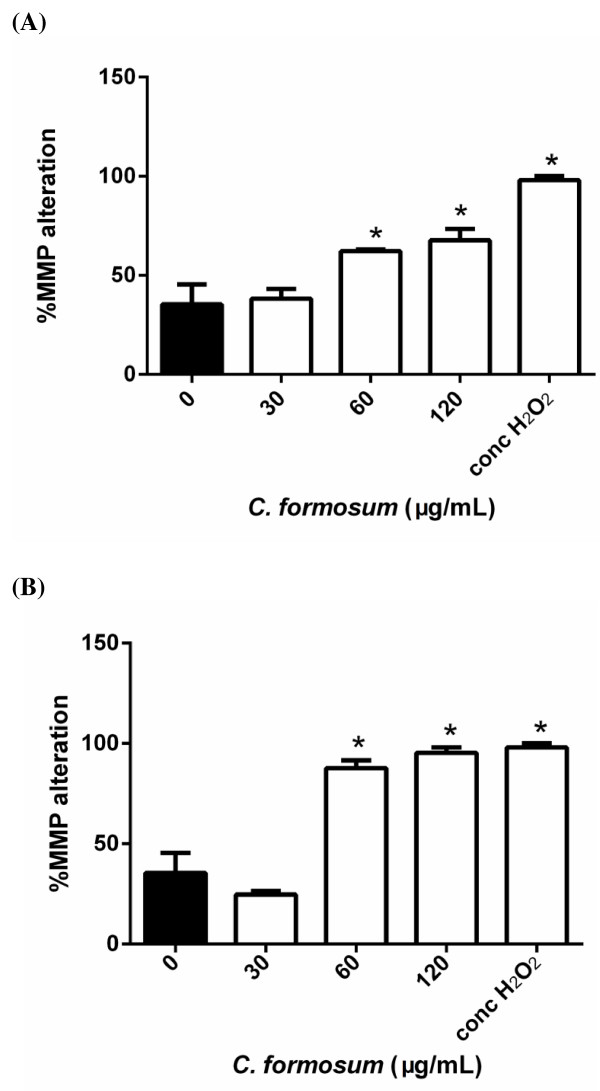
**Mitochondrial membrane potential in HepG2 cells exposed to CF extract at various concentrations for (A) 12 h and (B) 24 h, respectively, analyzed by flow cytometry.** *P < 0.05 significantly different from untreated cells.

MMP changes after cells were exposed to the CF extract at various concentrations were observed. The alteration of MMP in the cells treated with 30 μg/mL CF extract compared to untreated cells was not significantly different at 12 and 24 h (*P* = 0.994 and *P* = 0.150, respectively). The MMP alteration in the cells treated with 60 and 120 μg/mL CF extract and 35% (v/v) H_2_O_2_ were significantly different from the untreated at 12 and 24 h (*P* < 0.001, one-way ANOVA) (Figure 5).

### Total phenolic content of the CF extract

The total phenolic content was estimated by a Folin-Ciocalteu colorimetric method by gallic acid as the standard phenolic compound [19]. The calibration curve of gallic acid showed a linear correlation for gallic acid concentrations between 25–200 μg/mL, with an *r*^*2*^ of 0.997. The total phenolic content was 5.36 mg GAE/g dry weight of CF extract.

## Discussion

The current treatment of HCC includes 5-fluorouracil [23], doxorubicin, mitomycin C [24-26], cisplatin [25], and melphalan [27,28]. The 50% ethanol-water extract of CF inhibited the proliferation rate of HCC cells with a higher SI than melphalan. Based on published guidelines, any extract that possesses potentially toxic side effects must have an IC_50_ of less than 100 μg/mL [9]. Our laboratory previously found that CF induced apoptosis in leukemia cancer U937 cells [8] and this study aims to investigate the anticancer activity of 50% ethanol-water extract of CF.

Apoptosis was observed in the action mechanism of anti-cancer drugs [5-8]. The failure to trigger apoptosis was related to drug resistance and tumorigenesis [4]. Apoptosis includes both intrinsic and extrinsic pathways [29], and can be observed by investigating DNA condensation, membrane blebbing, and apoptotic body formation by staining cells with DAPI or Hoechst 3332 dye and detecting morphological changes under an inverted fluorescence microscope [29-31]. DNA fragmentation has also been investigated by 2D gel electrophoresis [30]. However, DNA fragmentation can also occur under non-apoptotic conditions [22].

Both apoptosis pathways are related to caspase activity, specifically caspase 3, 8, and 9 activity [29,32,33], that can be used to classify the extrinsic and intrinsic pathways [31]. The intrinsic or mitochondrial pathway is caused by nuclear DNA damage, resulting in alterations in the MMP, and release of cytochrome c into the cytosol. Ultimately, caspase 9 is also activated [31]. In the present study, the DNA condensation (Figure 2), MMP changes (Figure 5), and caspase 9 activation (Figure 4C and F) were observed after exposure of HepG2 cells to the CF extract.

The caspase 8 is the key caspase for the extrinsic pathway of apoptosis [34]*.* This extrinsic pathway of apoptosis occurred extrinsically from interactions of a death receptor with death ligands. In this study, the caspase 8 activity in HepG2 cell was increased after exposure to CF extract (Figure 4B and E).

Activation of caspase 8 and 9 also activates the executioner caspase 3/7, which can cleave several intracellular proteins and leads to cell disruption, including apoptotic body formation, chromosome breakage, and DNA fragmentation [30]. In the present study, CF induced the late stages of apoptosis characterized by an increase in caspase 3/7 activity (Figure 4A and D), as evidenced by apoptotic body formation (Figure 2) and DNA fragmentation (Figure 3). The induction of apoptosis by CF was clearly demonstrated by caspase activity in both the extrinsic and intrinsic pathways. Further studies of the crude CF extract effects on cancer cell lines are necessary to isolate the phytoconstituents contributing to the anticancer activity.

Previously, benzophenone glycosides that inhibited leukemia L1210 viability [35] were identified in a 60% ethanol extract from CF stems [10,36]. Xanthones, anthraquinones, and cochinchinone were identified in dichloromethane extracts of CF roots and bark [13,37], and quercetin, xanthones, and mangiferin were found in CF leaves and stem [38]. The bioactive compounds possessing anticancer activity in CF were cochinchinone A [39,40], macluraxanthon [41], norcowanin [42,43], and brasilixanthone [44]. Other activities were also reported for the dichloromethane extract, in which dulcisxanthone B had a DPPH-scavenging effect and inhibited lipid peroxidation [45].

The total phenolic content in the 50% ethanol-water extract of CF was 5.36 mg GAE/g dry weight of material. A previous report defined a high phenolic content as 20 mg GAE/g dry weight [46], based on the Folin-Ciocalteu method. Thus, the total content of the CF extract found in the present study would be considered low.

## Conclusions

CF extract induced a caspase-dependent apoptosis in HepG2 cell.

## Abbreviations

CF: *Cratoxylum formosum* (Jack) Dyer ssp. *pruniflorum* (Kurz) Gogel.; DAPI: 4′,6-diamidino-2-phenylindole; DiOC6: 3,3′-dihexyloxacarbocyanine iodide; DMEM: Dulbecco’s modified Eagle’s medium; DMSO: Dimethyl sulfoxide; FBS: Fetal bovine serum; GC-MS: Gas chromatography/mass spectrometry; HCC: Hepatocellular carcinoma; MMP: Mitochondrial membrane potential; NR: Reutral red; PBS: Phosphate-buffered saline; SI: Selectivity index; UV: Ultraviolet; RLU: Relative luminescence unit.

## Competing interests

The authors declare that they have no conflict of interests.

## Authors’ contributions

NW conceived and designed the study. AN performed the experiments and statistical analysis. NW and AN wrote the manuscript. SB reviewed the literature, revised the manuscript and coordinated the study. All the authors read and approved the final version of the manuscript.
